# Lookback Option Pricing with Fixed Proportional Transaction Costs under Fractional Brownian Motion

**DOI:** 10.1155/2014/746196

**Published:** 2014-11-03

**Authors:** Jiao-Jiao Sun, Shengwu Zhou, Yan Zhang, Miao Han, Fei Wang

**Affiliations:** College of Sciences, China University of Mining and Technology, Xuzhou 221116, China

## Abstract

The pricing problem of lookback option with a fixed proportion of transaction costs is investigated when the underlying asset price follows a fractional Brownian motion process. Firstly, using Leland's hedging method a partial differential equation satisfied by the value of the lookback option is derived. Then we obtain its numerical solution by constructing a Crank-Nicolson format. Finally, the effectiveness of the proposed form is verified through a numerical example. Meanwhile, the impact of transaction cost rate and volatility on lookback option value is discussed.

## 1. Introduction

In 1985, the problems of option pricing and replication with transaction costs were firstly examined by Leland [1]. Because of infinite variance of geometric Brownian motion process, transaction costs will become infinite, thus the arbitrary argument used by Black-Scholes to price options can no longer be used. Barles and Soner [2] considered the difference between the maximum utility from final wealth when there is no option liability and when there is such a liability. They derived the nonlinear Black-Scholes equation satisfied by the option value by using the utility maximization theory. Considering both transaction costs and the risk from a volatile portfolio, in 2005, Jandačka and Ševčovič [3] got another more complicated model. The influence of paying transaction costs and discontinuous change of stock price on option value was studied by Amster et al. [4]. Under a stochastic volatility process, Mariani et al. [5] investigated the option pricing problem with transaction cost and derived the pricing model. Meanwhile they got numerical solutions by using the finite difference method.

As a kind of exotic options, the holder of lookback option could look back on the evolution process of underlying assets during the life of option at maturity. It is one kind of path-dependent options where the payoff is based on the maximum or the minimum of the underlying asset price during the drift of the option. In 1979, the lookback option pricing formula was firstly given by Goldman et al. [6]. Then the pricing formula was extended by Conze and Viswanathan [7]. They obtained explicit formulas of various European lookback options and also provided some results for the American counterparts by means of probability method. With underlying asset price following geometric Brownian motion, analytic expressions of discrete lookback option value were presented by Heynen and Kat [8]. Many empirical studies have shown that distribution of asset price is not entirely lognormal, and the probability density function of its logarithm yield tends to have the feature of “fat-tailedness,” so geometric Brownian motion processes do not accord with the realistic environment. For characteristics of self-similarity, fat tails, and long-term dependence, fractional Brownian motion (fBm) becomes an effective tool to describe the stock price process instead of geometric Brownian motion. fBm is neither a Markov process nor a semimartingale, so stochastic analysis theory for semimartingale cannot be applied directly. In incomplete markets, arbitrage opportunities will exist. A considerable number of arbitrage strategies for fBm have been provided by Rogers [9], Shiryaev [10], Salopek [11], and Cheridito [12]. In 2012, Gu et al. [13] dealt with the problem of discrete time option pricing in the presence of transaction costs by a time-changed geometric fractional Brownian motion model. Through a mean self-financing delta-hedging argument, they obtained the pricing formula for the European call option in discrete time setting. Feng [14] discussed the lookback option under fBm model. They derived the explicit solutions of lookback put option pricing formula.

In this paper, we will firstly give the price dynamics model under fBm process. Then a three-dimensional nonlinear mathematical pricing model for lookback put option value with fixed transaction costs will be established. We reduced it to a two-dimensional model through variable substitution. But the reduced model is still nonlinear, so it is not easy to get the analytic solution. So we get its numerical solution by constructing a Crank-Nicolson numerical scheme for the transformed model. Finally, we analyze the effectiveness of the numerical scheme and the influence of parameters on lookback put option value.

## 2. The Model Dynamics

**Definition 1 .**

Let *H* be a constant belonging to (0,1). A fractional Brownian motion (*B* ^*H*^(*t*))_*t*≥0_ of Hurst index *H* is a continuous and centered Gaussian process with covariance function

(1) $$\begin{array}{c} E\left[B^{H}\left(t\right)B^{H}\left(s\right)\right]=\frac{1}{2}\left(t^{2H}+s^{2H}-{\left|t-s\right|}^{2H}\right). \end{array}$$

For *H* = 0.5, the fBm is then a standard Brownian motion. By Definition 1 we obtain that a standard fBm *B* ^*H*^(*t*) has the following properties.

(i) *B* ^*H*^(0) = 0 and *E*[*B* ^*H*^(*t*)] = 0 for all *t* ≥ 0;
(ii) *B* ^*H*^(*t*) has homogeneous increments; that is, *B* ^*H*^(*t* + *s*) − *B* ^*H*^(*s*) has the same law of *B* ^*H*^(*t*) for *s*, *t* ≥ 0;
(iii) *B* ^*H*^(*t*) is a Gaussian process and *E*[(*B* ^*H*^(*t*))^2^] = *t* ^2*H*^, *t* ≥ 0, for all *H* ∈ (0,1);
(iv) *B* ^*H*^(*t*) has continuous trajectories.

The fBm has long-term dependency for *H* ∈ (0.5,1) and is antisustainable for *H* ∈ (0,0.5). For *H* ≠ 0.5, it is neither a Markov process nor semimartingale, so the classical Itô's calculus cannot be used to define a fully stochastic calculus for fBm. Lin [15] and Decreusefond and üstünel [16] developed the stochastic calculus of variations under fBm model, but Shiryaev [10] investigated that there exists arbitrage chance in the market. Hu and Øksendal [17] presented the fractional Itô's calculus based on the Wick integration driven by fBm. Bender [18] constructed fractional Itô's formula with this type of calculus. For more details about fBm, interested readers may refer to Hu and Peng [19].

Consider a financial market with two primitive securities, namely, a risky asset *S*_*t*_ and a risk-free bond *B*_*t*_. We will need the following assumptions.

(i) The securities trading is carried out continuously with short selling allowed;
(ii) Suppose *v*_*t*_ shares of the traded stock are bought (*v*_*t*_ > 0) and sold (*v*_*t*_ < 0) at the price *S*_*t*_. Then a proportion of transaction cost is given by *κ* | *v*_*t*_ | *S*_*t*_ in either buying or selling, where *κ* > 0 is the fixed transaction cost rate.
(iii) There exists no arbitrage opportunities and the portfolio's expected rate is the risk-free interest rate *r*, where *r* is a constant.
(iv) The portfolio is revised every time interval *δt*, where *δt* is a finite and fixed time step.

The price process *B*_*t*_ of risk-free debt evolves over time as

(2) $$\begin{array}{c} B_{t}=e^{rt},\;\;B_{0}=1. \end{array}$$

Suppose that the price process *S*_*t*_ of the risky share evolves over time as

(3) $$\begin{array}{c} dS_{t}=rS_{t}dt+\sigma S_{t}dB_{H}\left(t\right),\;S_{0}=s>0, \end{array}$$

where *σ* indicates the volatility of the stock price.

## 3. The Valuation of Lookback Put Option with Transaction Costs under fBm

In this section, we adopt Leland hedging strategy to derive a model for lookback option price. The following lemma gives Fractional Itô's differential rule which takes great value in deriving the formula.

**Lemma 2 (see [18]).**

Consider the stochastic differential equation

(4) $$\begin{array}{c} dS_{t}=\mu \left(t,\omega \right)dt+\sigma \left(t,\omega \right)dB_{H}\left(t\right). \end{array}$$

Here for *t* ≥ 0, *μ*(*t*, *ω*) and *σ*(*t*, *ω*):[0, *T*] × *R* → *R* are two stochastic processes. Assume that a two-variable function *f*(*t*, *S*_*t*_):[0, *T*] × *R* ^+^ → *R* has uniformly continuous partial derivatives ∂*f*/∂*t*, ∂*f*/∂*S*_*t*_, ∂^2^ *f*/∂*S*_*t*_^2^. Then for *H* ∈ (0,1), we have

(5) dft,St=∂∂tf(t,St)dt+∂∂Stf(t,St)μ(t,ω)dt +∂∂Stft,Stσt,ωdBHt +H∂2∂St2ft,Stσ2t,ωt2H−1dt.

Consider a European-type lookback strike put option. Let *V*_*t*_ = *V*(*S*_*t*_, *J*_*t*_, *t*) which denotes the value of the option at time *t* The corresponding payoff *J*_*t*_ = max⁡_0≤*u*≤*t*_⁡*S*_*u*_, where *S*_*t*_ satisfies (3). The following theorem gives the model for the value of lookback put option with transaction costs.

**Theorem 3 .**

The pricing model for the value of lookback put option with transaction costs under fractional Brownian motion process is given as

(6) $$\begin{array}{c} \frac{\partial V_{t}}{\partial t}+\frac{1}{2}{\tilde{\sigma }}^{2}S_{t}^{2}\frac{\partial^{2}V_{t}}{\partial S_{t}^{2}}+rS\frac{\partial V_{t}}{\partial S_{t}}-rV_{t}=0,\\ V\left(S_{T},J_{T},T\right)=J_{T}-S_{T},\;\left(0\leq S_{T}\leq J_{T}<\infty \right),\\ {\left.\frac{\partial V_{t}}{\partial J_{t}}\right|}_{S_{t}=J_{t}}=0.\;\;\left(0\leq t\leq T\right), \end{array}$$

where ${\tilde{\sigma }}^{2}=2\sigma^{2}\left(Ht^{2H-1}-Le\left(H\right)\mathrm{sign}(V_{SS})\right)$, $\;Le\left(H\right)=\sqrt{2/\pi }(\kappa /\sigma ){\left(\delta t\right)}^{H-1}$, *δt* is the hedging time interval.

**Proof**

For the option purchaser, a hedging portfolio Π_*t*_ is constructed: one long position of lookback put option and short Δ_*t*_ shares of stock; then the value of the portfolio at the current time *t* is Π_*t*_ = *V*_*t*_ − Δ_*t*_ *S*_*t*_. Assume that the trade occurs only at times *t* and *t* + *δt* where *δt* denotes the hedging time interval. Then over a small time interval *δt*, the change in value of the portfolio will be

(7) $$\begin{array}{c} \delta \Pi_{t}=\delta V_{t}-\Delta_{t}\delta S_{t}-\kappa \left|v_{t}\right|S_{t}. \end{array}$$

The stochastic partial differential equation (3) can be shown as

(8) $$\begin{array}{c} \delta S_{t}=rS_{t}\delta t+\sigma S_{t}\delta B_{H}\left(t\right). \end{array}$$

By using discrete form of Lemma 2, we can get

(9) δVt=∂Vt∂t+rSt∂Vt∂St+Hσ2t2H−1St2∂2Vt∂St2δt +σSt∂Vt∂StδBHt+∂Vt∂JtδJt.

Substituting (9) into (7) yields

(10) δΠt=∂Vt∂t+Hσ2St2t2H−1∂2Vt∂St2δt+∂Vt∂St−ΔtδSt +∂Vt∂JtδJt−κvtSt.

In order to eliminate risk, let Δ_*t*_ = ∂*V*_*t*_/∂*S*_*t*_. So in the same interval *δt*, the traded shares of stock are

(11) vt=∂V∂St(St+δt,Jt+δt,t+δt)−∂V∂St(St,Jt,t)≈∂2V∂St2(St,Jt,t)σStδBH(t)+∂2Vt∂St∂JtδJt.

Since *J*_*t*_ is not differentiable, we need to approximate it as

(12) $$\begin{array}{c} J_{n}\left(t\right)={\left[\frac{1}{t}\overset{t}{\underset{0}{\int }}{\left(S_{\tau }\right)}^{n}d\tau \right]}^{1/n},\;\left(0\leq \tau \leq t\right); \end{array}$$

then

(13) $$\begin{array}{c} nJ_{n}^{n-1}\left(t\right)\frac{dJ_{n}}{dt}=\frac{S_{t}^{n}-J_{n}^{n}\left(t\right)}{t},\\ \underset{n\to \infty }{\lim}\;J_{n}\left(t\right)=\underset{0\leq \tau \leq t}{\max}\;S_{\tau }=J_{t}. \end{array}$$

We use *J*_*n*_(*t*) instead of path dependence variable *J*_*t*_; since *S*_*t*_ ≤ *J*_*t*_, we can get

(14) $$\begin{array}{c} \frac{{\left(S_{t}/J_{n}\right)}^{n-1}S_{t}-J_{n}}{nt}⟶0\;\left(n⟶\infty \right). \end{array}$$

Consequently, from (11)

(15) $$\begin{array}{c} v_{t}\approx \frac{\partial^{2}V}{\partial S_{t}^{2}}\left(S_{t},J_{t},t\right)\sigma S_{t}\delta B_{H}\left(t\right). \end{array}$$

The transaction costs is as follows:

(16) κvtSt=κ∂2V∂St2(St,Jt,t)σSt2δBH(t)=κσSt2∂2V∂St2(St,Jt,t)δBH(t).

According to the Definition 1, we know that

(17) $$\begin{array}{c} \delta B_{H}\left(t\right)=B_{H}\left(t+\delta t\right)-B_{H}\left(t\right)\sim N\left(0,{\left(\delta t\right)}^{2H}\right). \end{array}$$

Therefore

(18) EκvtSt=EκσSt2∂2V∂St2(St,Jt,t)δBH(t)=2πκσSt2δtH∂2Vt∂St2+Oδt.

From the assumption (iii), we have *E*(*δ*Π_*t*_) = *r*Π_*t*_ *δt*. Thus

(19) rVt−ΔtStδt=∂Vt∂t+Hσ2St2t2H−1∂2Vt∂St2δt +∂Vt∂JtδJt−2πκσSt2δtH∂2Vt∂St2.

Consequently

(20) ∂Vt∂t+Hσ2St2t2H−1∂2Vt∂St2−2πκσSt2δtH−1∂2Vt∂St2  +rSt∂Vt∂St−rVt=0.

The above equation can be simplified as

(21) $$\begin{array}{c} \frac{\partial V_{t}}{\partial t}+\frac{1}{2}{\tilde{\sigma }}^{2}S_{t}^{2}\frac{\partial^{2}V_{t}}{\partial S_{t}^{2}}+rS_{t}\frac{\partial V_{t}}{\partial S_{t}}-rV_{t}=0. \end{array}$$

Here

(22) $$\begin{array}{c} Le\left(H\right)=\sqrt{\frac{2}{\pi }}\frac{\kappa }{\sigma }{\left(\delta t\right)}^{H-1},\\ {\tilde{\sigma }}^{2}=2\sigma^{2}\left(Ht^{2H-1}-Le\left(H\right)\mathrm{sign}\left(V_{SS}\right)\right). \end{array}$$

The boundary condition is

(23) $$\begin{array}{c} {\left.\frac{\partial V_{t}}{\partial J_{t}}\right|}_{S_{t}=J_{t}}=0, \end{array}$$

whose financial significance is that the value of option is not sensitive to the maximum when the underlying asset price reaches the maximum [20]. According to the definition of the lookback put option with floating strike price, the formula can be given as

(24) $$\begin{array}{c} V\left(S_{T},J_{T},T\right)=J_{T}-S_{T}. \end{array}$$

Hence the result is proved.

Since the parameter $\tilde{\sigma }$ is nonlinear in the model (6), it's difficult to get its analytic solution. Then we will give its numerical scheme in the next section.

## 4. Crank-Nicolson Scheme

Firstly we should reduce the three-dimensional nonlinear mathematical model (6) into a corresponding two-dimensional model so that it can be solved more easily. Let

(25) $$\begin{array}{c} x=\ln\frac{J_{t}}{S_{t}},\;\;V\left(S_{t},J_{t},t\right)=S_{t}u\left(x,\tau \right),\;\tau =T-t; \end{array}$$

consequently

(26) ∂u∂τ+r+12σ^2∂u∂x−12σ^2∂2u∂x2=0,hhhhhhhh0<x<∞,  0≤τ≤T,ux,ττ=0=ex−1, 0≤x<∞,  ∂u∂xx=0=0, 0≤τ≤T,

where

(27) $$\begin{array}{c} {\hat{\sigma }}^{2}=2\sigma^{2}\left(H{\left(T-\tau \right)}^{2H-1}-Le\left(H\right)\mathrm{sign}\left(u_{xx}-u_{x}\right)\right). \end{array}$$

Then the Crank-Nicolson scheme will be constructed for the nonlinear problem (26). Consider the problem to a finite domain *Ω* = [0, *x*_max⁡_]×[0, *T*]. Discrete it using the following method and construct a group of grid points (*x*, *τ*) = (*x*_*i*_, *τ*_*n*_). Let

(28) ui,n=uxi,τn,  xi=ih,  tn=nk,hhhli=0,1,2,…,M, n=0,1,2,…,N.

With the time step *k* = *T*/*N* and spatial step *h* = *x*_max⁡_/*M*.

Time derivatives and spatial derivatives are, respectively, approximated by difference method

(29) ∂u∂τ(xi,τn)≈uin+1−uink,∂u∂x(xi,τn)≈12ui+1n−ui−1n2h+ui+1n+1−ui−1n+12h,∂2u∂x2(xi,τn)  ≈12ui+1n−2uin+ui−1nh2+ui+1n+1−2uin+1+ui−1n+1h2.

Substituting the above equations into the model (26) gives standard Crank-Nicolson scheme. Since the corrected volatility (27) has first- and second-order derivatives, its solution can be obtained through nonlinear iteration. But it will consume too much time. We will use standard central difference scheme to approximate it. Hence we get the new numerical scheme as follows:

(30) uin+1−uink  −12ρ1,inui+1n−2uin+ui−1nh2+ui+1n+1−2uin+1+ui−1n+1h2  +12ρ2,inui+1n−ui−1n2h+ui+1n+1−ui−1n+12h=0,

(31) $$\begin{array}{c} \rho_{1,i}^{n}=2\sigma^{2}\left(H{\left(T-\tau_{n}\right)}^{2H-1}-Le\left(H\right)\mathrm{sign}\left(\Delta_{i}^{n}-\nabla_{i}^{n}\right)\right),\\ \rho_{2,i}^{n}=r+\rho_{1,i}^{n}, \end{array}$$

with

(32) $$\begin{array}{c} \Delta_{i}^{n}=\frac{u_{i+1}^{n}-2u_{i}^{n}+u_{i-1}^{n}}{h^{2}},\;\;\nabla_{i}^{n}=\frac{u_{i+1}^{n}-u_{i-1}^{n}}{2h}. \end{array}$$

So the numerical scheme equals

(33) ainui+1n+1+binuin+1+cinui−1n+1=αinui+1n+βinuin+γinui−1n,hhhhhhhhhhhhhhhhhhhhhhhhli=1,2,…,M−1,

where

(34) αin=k2h2ρ1,in−k4hρ2,in,βin=1−kh2ρ1,in,γin=k2h2ρ1,in+k4hρ2,in,ain=−k2h2ρ1,in+k4hρ2,in,bin=1+kh2ρ1,in,cin=−k2h2ρ1,in−k4hρ2,in.

The format (33) is a six-point implicit scheme, whose local truncation error is *O*(*h* ^2^ + *k* ^2^). Only a little computation can achieve satisfactory accuracy. For numerical convenience, we define a vector *u* ^*n*^ = [*u*_1_^*n*^, *u*_2_^*n*^,…, *u*_*M*−1_^*n*^]^*T*^. Then the format (33) can be given as below:

(35) $$\begin{array}{c} A\left(n\right)u^{n+1}=B\left(n\right)u^{n}+p^{n},\;n=0,1,2,\ldots ,N-1. \end{array}$$

Here

(36) $$\begin{array}{c} A\left(n\right)=\left[\begin{array}{cccccc} b_{1}^{n} & a_{1}^{n} & 0 & 0 & \cdots & 0\\ c_{2}^{n} & b_{2}^{n} & a_{2}^{n} & 0 & \cdots & 0\\ 0 & c_{3}^{n} & b_{3}^{n} & a_{3}^{n} & \cdots & 0\\ & & \ddots & \ddots & \ddots & \\ 0 & 0 & \cdots & c_{M-2}^{n} & b_{M-2}^{n} & a_{M-2}^{n}\\ 0 & 0 & \cdots & 0 & c_{M-1}^{n} & b_{M-1}^{n} \end{array}\right],\\ B\left(n\right)=\left[\begin{array}{cccccc} \beta_{1}^{n} & a_{1}^{n} & 0 & 0 & \cdots & 0\\ \gamma_{2}^{n} & \beta_{2}^{n} & a_{2}^{n} & 0 & \cdots & 0\\ 0 & \gamma_{3}^{n} & \beta_{3}^{n} & a_{3}^{n} & \cdots & 0\\ & & \ddots & \ddots & \ddots & \\ 0 & 0 & \cdots & \gamma_{M-2}^{n} & \beta_{M-2}^{n} & \alpha_{M-2}^{n}\\ 0 & 0 & \cdots & 0 & \gamma_{M-1}^{n} & \beta_{M-1}^{n} \end{array}\right],\\ p^{n}={\left[\gamma_{1}^{n}u_{0}^{n}-c_{1}^{n}u_{0}^{n+1},0,\ldots 0,\alpha_{M-1}^{n}u_{M}^{n}-a_{M-1}^{n}u_{M}^{n+1}\right]}^{T}. \end{array}$$

We also need to know the value of the boundary points *u*_*M*_^*n*^ and *u*_0_^*n*^. So discrete the boundary conditions by second-order Gear formula.

Hence

(37) $$\begin{array}{c} {\left.\frac{\partial u}{\partial x}\right|}_{x=0}=\frac{3u_{0}^{n}-4u_{1}^{n}+u_{2}^{n}}{2h}=0,\;\text{that is,}\;\;u_{0}^{n}=\frac{4u_{1}^{n}-u_{2}^{n}}{3}. \end{array}$$

When the price of underlying asset *S*_*t*_ reaches 0, the lookback put option must be exercised. Therefore another boundary condition can be given as

(38) $$\begin{array}{c} V\left(0,J_{t},t\right)=e^{-r(T-t)}J_{t}. \end{array}$$

According to variable substitution (25), we have

(39) $$\begin{array}{c} u_{M}^{n}=e^{-r\tau }. \end{array}$$

Substituting the boundary conditions (37) and (39) into (35) and using chasing method gives this format's solution. The value of the lookback put option at the starting time is *su*_0_^*N*^. It can be seen that it does not contain the variable *J*_*t*_ in the new numerical scheme so that it avoids the difficulty of determining the variable *J*_*t*_ in the iterative process.

## 5. Numerical Example

In this section, we will firstly study the convergence of the numerical scheme to explain its validity and discuss the influence of various parameters on the lookback option's value. For simplicity, we will only consider valuation of lookback put option for *H* ∈ (0.5,1).

**Example 1 .**

Consider a European put option in half a year when the underlying asset is a stock, whose price follows Fractional Brown Motion (3), and the option value is satisfied by the model (6). Suppose that the stock price is $30, the volatility is 20% per annum, the risk-free interest rate is 5%, the Hurst parameter is 0.6, the hedging time interval is 0.005, and the number of time steps is taken as 100, spatial steps taken as 300 in the numerical scheme (35). With our usual notation, this means that

(40) $$\begin{array}{c} T=0.5,\;\;\;r=0.05,\;\;\sigma =0.2,\\ S_{t}=30,\;\;\;H=0.6,\;\;\delta t=0.005,\\ M=300,\;\;N=100. \end{array}$$

For different values of Hurst parameter, Table 1 shows the price for lookback put option with transaction cost through Crank-Nicolson numerical approach and proves its convergence of this method. From Table 1, we can also see that, with the increase of Hurst parameter, the magnitude of decrease for lookback put option value is increasing. This implies the self-similarity of fBm model for *H* ∈ (0.5,1).

**Figure 1:**
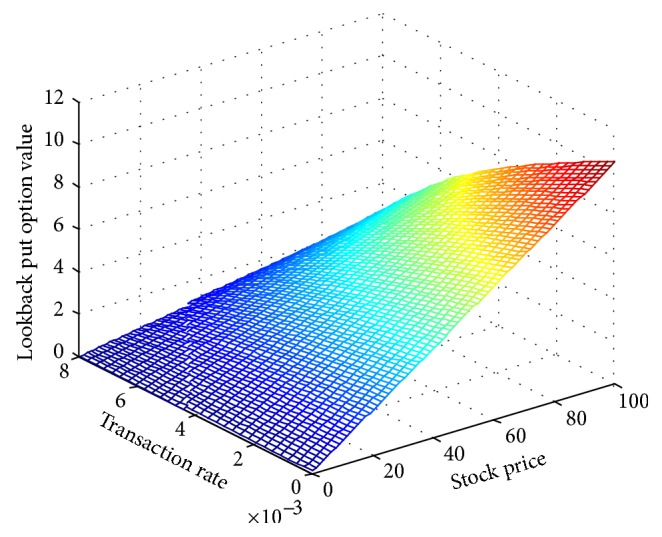
Lookback put option value along with transaction costs and stock price.

Then we study the influence of transaction cost rates on lookback put option value. Other values of parameters remain unchanged.  Figure 1 displays that the value of the option is decreasing with the increase of transaction costs. This is mainly because in this paper, for the long position of options, we only consider the purchase price. Transaction rates' increase makes a unit of an asset's cost increase, directly leading to the option hedging costs' raising. So option value will decrease. Besides, with the initial stock price increased, the lookback put option value increases.

**Figure 2:**
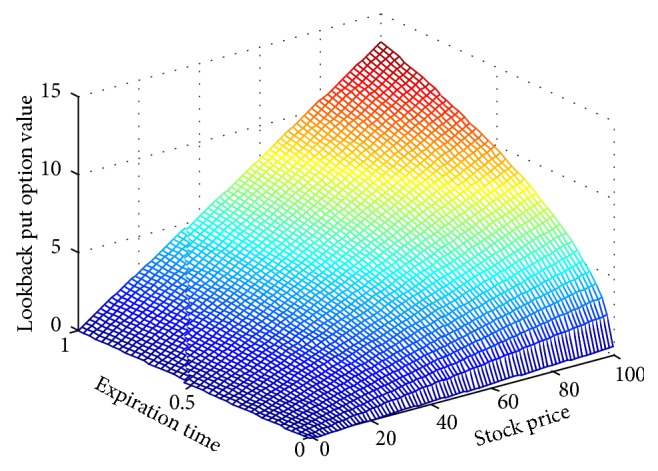
Lookback put option value along with expiration time and stock price.

Figure 2 shows the effect of expiration time *T*and stock price *S*_*t*_ on lookback put option value. From it we can see that longer maturity time can produce larger option value. Moreover, when the expiration time is shorter, it grows more obviously. This is mainly because that the option value increases over time.

**Figure 3:**
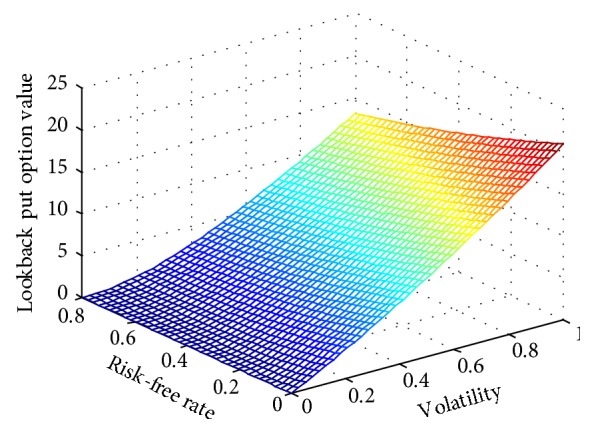
Lookback put option value along with risk-free rate and volatility.

Then we discuss the impact of risk-free rate and volatility change on lookback put option value. From Figure 3, it can be seen that the option value decreases with risk-free rate increasing. One factor is that the expected rate of return rises as risk-free rate grows. Another factor is that risk-free rate's growth leads to the present value of future cash flows dropping. On the other hand, the larger the volatility, the larger the option value. It is mainly because that volatility's increase amplifies the probability of making large gains in the future.

Finally, we illustrate the advantages of the model in this paper from the data in financial market. Taking China Guodian warrants for example, we will use the new model presented in this paper and Black-scholes model to reprice the warrants and obtain the error between theoretical and empirical price to reflect the rationality of the model proposed. China Guodian warrants was listed on May 22, 2008, and exercised on May 21, 2010, with the strike price 7.47 yuan and the right ratio 1.00. From the historical data of the stock price for China Guodian power (600795) during the time interval from March 18, 1997, to November 1, 2009, it can be estimated that the volatility *σ* = 0.5937. Assume that interest rate *r* = 2% and transaction cost rate *κ* = 0.001.

Table 2 below shows the statistical characteristics of the logarithmic rate of return for China Guodian warrants. It can be observed that the value of kurtosis for the warrants is greater than that for the standard normal distribution and the probability to obtain corresponding statistic *J*-*B* is less than 0.05, which indicates share earnings is distributed the feature aiguille large remaining part.

**Figure 4:**
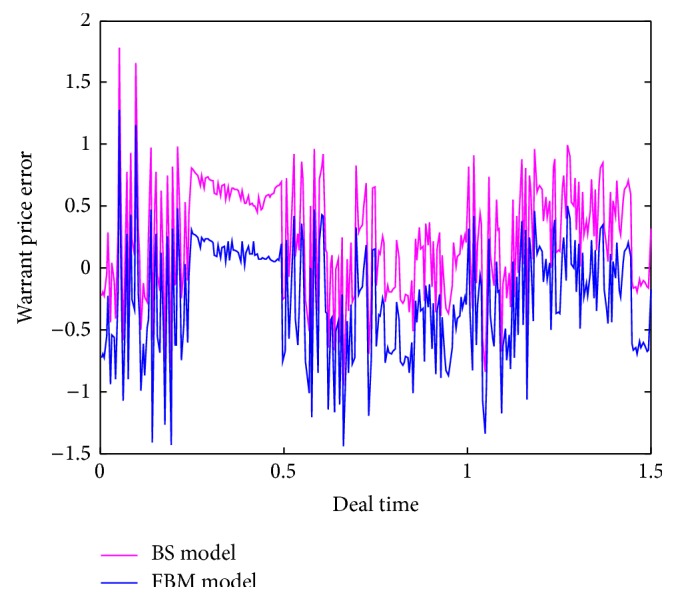
Comparison of pricing errors of China Guodian warrants under two different models.

By comparison between theoretical and empirical prices, Figure 4 shows pricing errors under the pricing model of lookback option with transaction costs based on fractional Brownian motion process (*H* = 0.7) and Black-Scholes model, respectively. As can be seen from Figure 4, the pricing errors under FBM model are significantly less than BS model. This indicates that the new model involved in this paper is more realistic.

## 6. Conclusions

In this paper, the lookback option pricing problem, with proportional transaction costs under the Fractional Brown Motion model, was studied. By using hedging principle, the nonlinear partial differential equations, satisfied by the option value, have been derived. As for the equations, we have constructed the Crank-Nicolson scheme and verified its effectiveness by using the Matlab software. It can be seen that the model we have built up is more in line with the reality of the market environment through Matlab software simulation.

## Figures and Tables

**Table 1:** Convergence for lookback put option with transaction cost in the fBm Process.

*H*	Number of time steps							
	100	300	500	1000	2000	3000	4000	5000
0.6	2.6325	2.6281	2.6271	2.6264	2.6261	2.6259	2.6259	2.6258
0.7	2.5247	2.5186	2.5174	2.5164	2.5159	2.5158	2.5157	2.5157
0.8	2.4008	2.3940	2.3926	2.3916	2.3910	2.3909	2.3908	2.3907
0.9	2.2697	2.2625	2.2610	2.2599	2.2594	2.2592	2.2591	2.2590

**Table 2:** The basic statistical characteristics of China Guodian warrants (data sources: China Tai'an database).

Statistics	Rates of return for China Guodian warrants
Mean value	−0.00025
Maximum	0.288731
Minimum	−0.15535
Variance	0.00168
Median	0.002021
Skewness	0.905533
Kurtosis	10.6741
*J*-*B* statistics	875.5861
Probability	0.001
